# Supplementary material for: Butyrate enhances Clostridioides difficile sporulation in vitro
Source: J Bacteriol. 2023 Sep 1;205(9):e00138-23. doi: 10.1128/jb.00138-23 (PMC10521354; doi:10.1128/jb.00138-23)
Supplement: Supplemental figures — All supplemental figures with their respective legends. [file jb.00138-23-s0001.pdf]

Supplemental Outline:

Figure S1. Butyrate does not significantly impact the growth of *C. difficile* strains R20291 or VPI10643. Growth curves ($\log_{10}(OD_{600})$) of *C. difficile* **A)** strain R20291 and **B)** strain VPI10463 over 24 hours in presence of 5 and 25 mM acetate, propionate, and butyrate in BHI.

Figure S2. Butyrate-induced growth inhibition is not impacted by pH. Growth of *C. difficile* strain 630 over 24 hours at pH 6.2 (panel 1), 7.2 (panel 2), and 8 (panel 3) in BHI supplemented with 5 or 25 mM acetate, propionate, and butyrate. P-value through Dunnett's test, *p-value < 0.05; **p-value < 0.01; ***p-value < 0.001.

Figure S3. Short chain fatty acids increase toxin production in *C. difficile* strains R20291 and VPI10463. Toxin activity ($\log_{10}$ of toxin mg/ml normalized to average $\log_{10}$(CFU/ml) of *C. difficile* strain **A**) R20291 and **B)** VPI10463 over 24 hours in BHI supplemented with 5 or 25 mM acetate, propionate, and butyrate as measured by an *in vitro* cell assay per log10 (CFU/ml). P-value through Dunnett's test, *p-value < 0.05; **p-value < 0.01; ***p-value < 0.001.

Figure S4. Butyrate increases the expression of carbohydrate metabolism genes. Heatmap of selected significant genes representing the $\log_2$FoldChange of growth in butyrate versus without butyrate in early- and late-log calculated using DESeq in R as described for RNASeq analysis in Materials and Methods. Significance ($p < 0.05$) was calculated using Wald's test; all genes listed are significantly over- or under-expressed over growth in BHI alone except *ccpA* (not significant).

Figure S5. Butyrate modulates select *C. difficile* carbohydrate metabolism and virulence regulators. Relative abundance of normalized transcript counts of select significant genes in

early- and late-log. Normalized transcripts were obtained using nnTransform in DESeq. The significance was calculated by one-way ANOVA between transcripts from BHI growth with or without butyrate *p-value < 0.05; **p-value < 0.01; ***p-value < 0.001

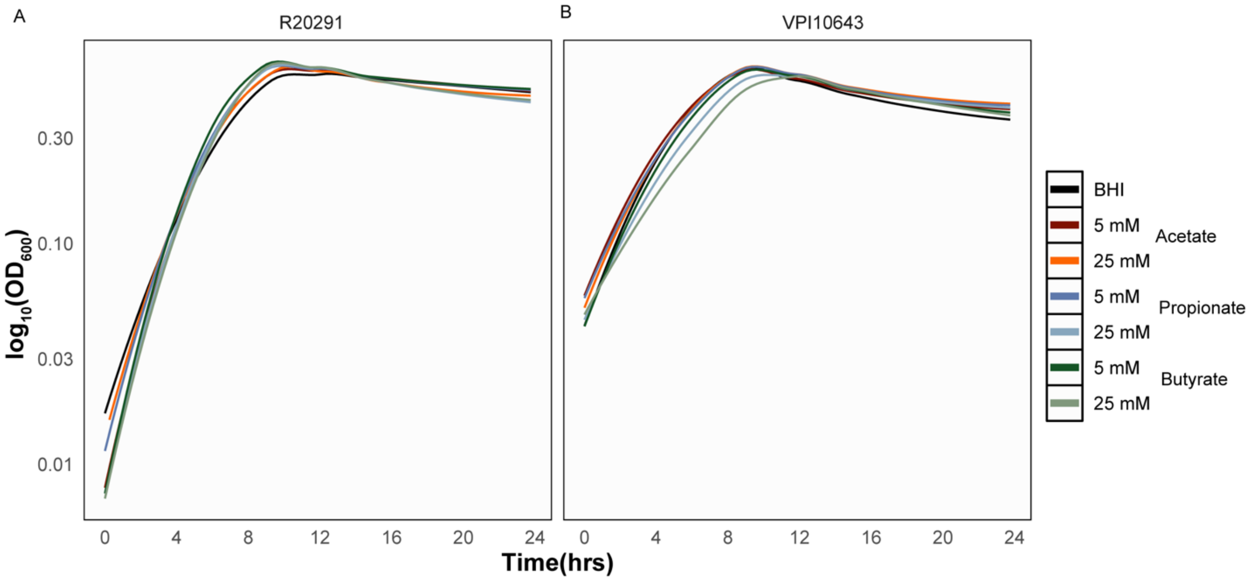

Figure S1. Butyrate does not significantly impact the growth of *C. difficile* strains R20291 or VPI10643. Growth curves ($\log_{10}(OD_{600})$) of *C. difficile* **A)** strain R20291 and **B)** strain VPI10463 over 24 hours in presence of 5 and 25 mM acetate, propionate, and butyrate in BHI.

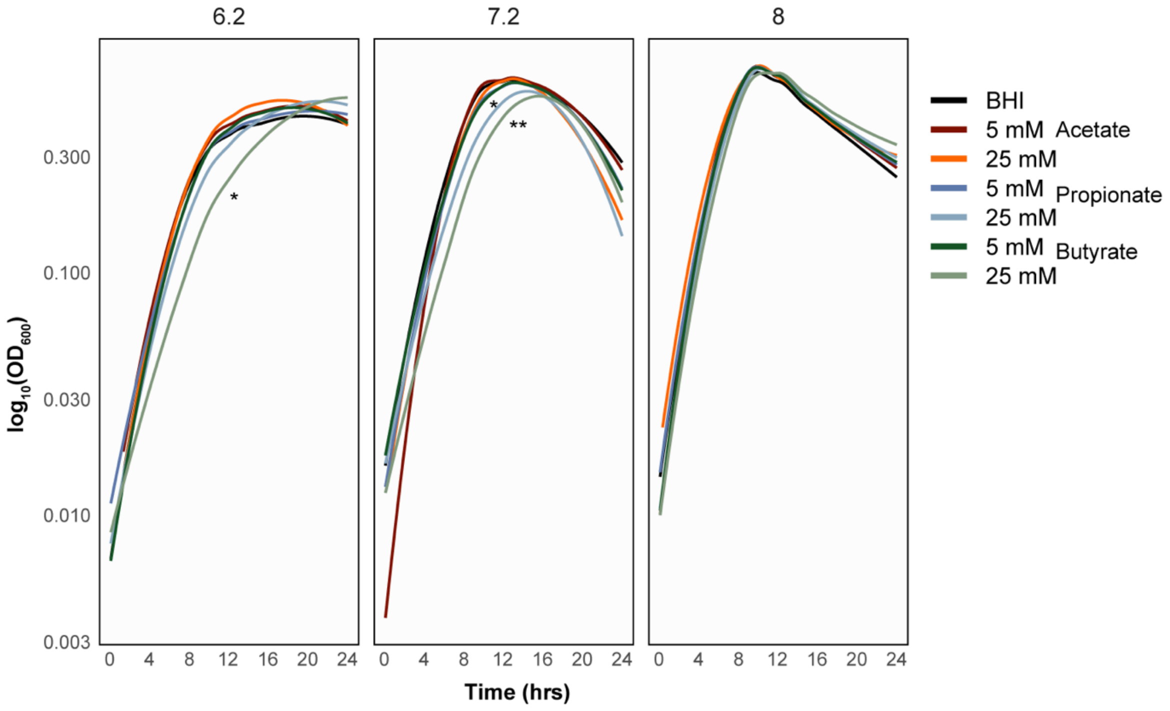

Figure S2. Butyrate-induced growth inhibition is not impacted by pH. Growth of *C. difficile* strain 630 over 24 hours at pH 6.2 (panel 1), 7.2 (panel 2), and 8 (panel 3) in BHI supplemented with 5 or 25 mM acetate, propionate, and butyrate. P-value through Dunnett's test, *p-value < 0.05; **p-value < 0.01; ***p-value < 0.001.

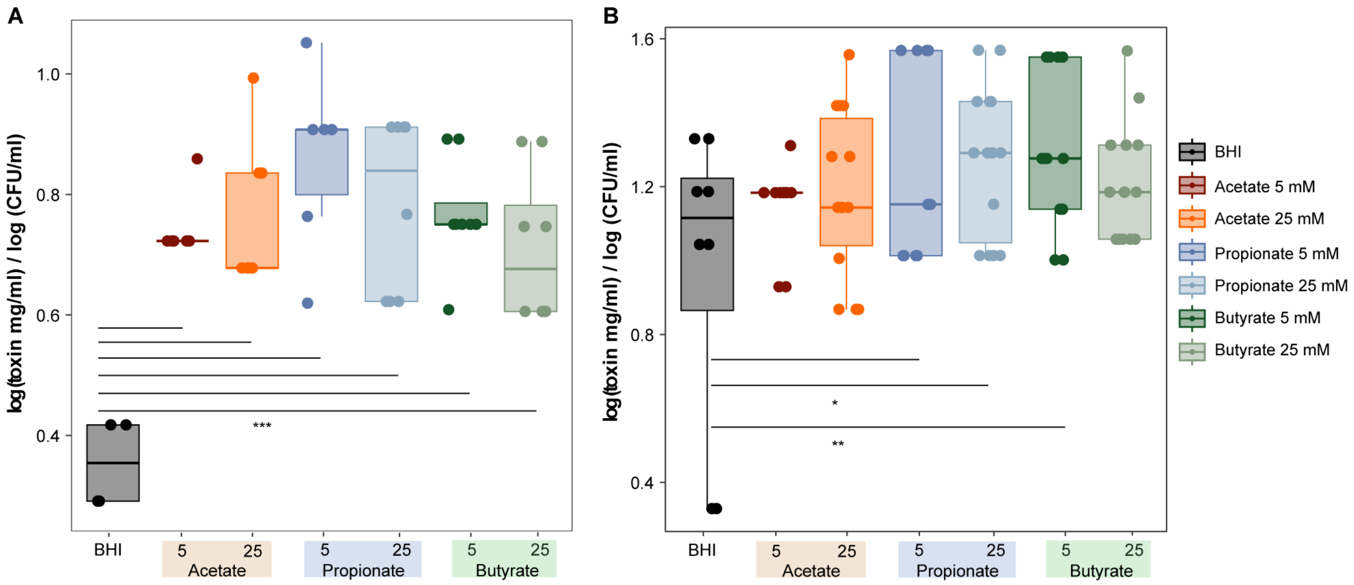

Figure S3. Short chain fatty acids increase toxin production in *C. difficile* strains R20291 and VPI10463. Toxin activity (log_{10} of toxin mg/ml normalized to average log_{10}(CFU/ml) of *C. difficile* strain **A**) R20291 and **B)** VPI10463 over 24 hours in BHI supplemented with 5 or 25 mM acetate, propionate, and butyrate as measured by an *in vitro* cell assay per log10 (CFU/ml). P-value through Dunnett's test, *p-value < 0.05; **p-value < 0.01; ***p-value < 0.001.

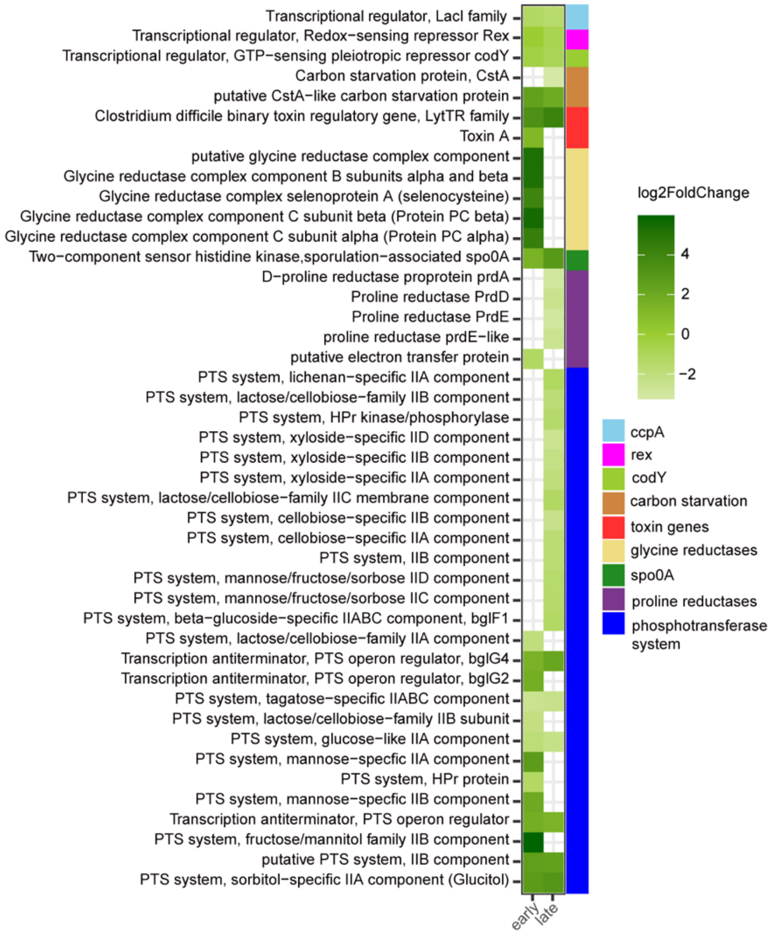

Figure S4. Butyrate increases the expression of carbohydrate metabolism genes.

Heatmap of selected significant genes representing the log_2FoldChange of growth in butyrate versus without butyrate in early- and late-log calculated using DESeq in R as described for RNASeq analysis in Materials and Methods. Significance ($p < 0.05$) was calculated using Wald's test; all genes listed are significantly over- or under-expressed over growth in BHI alone except *ccpA* (not significant).

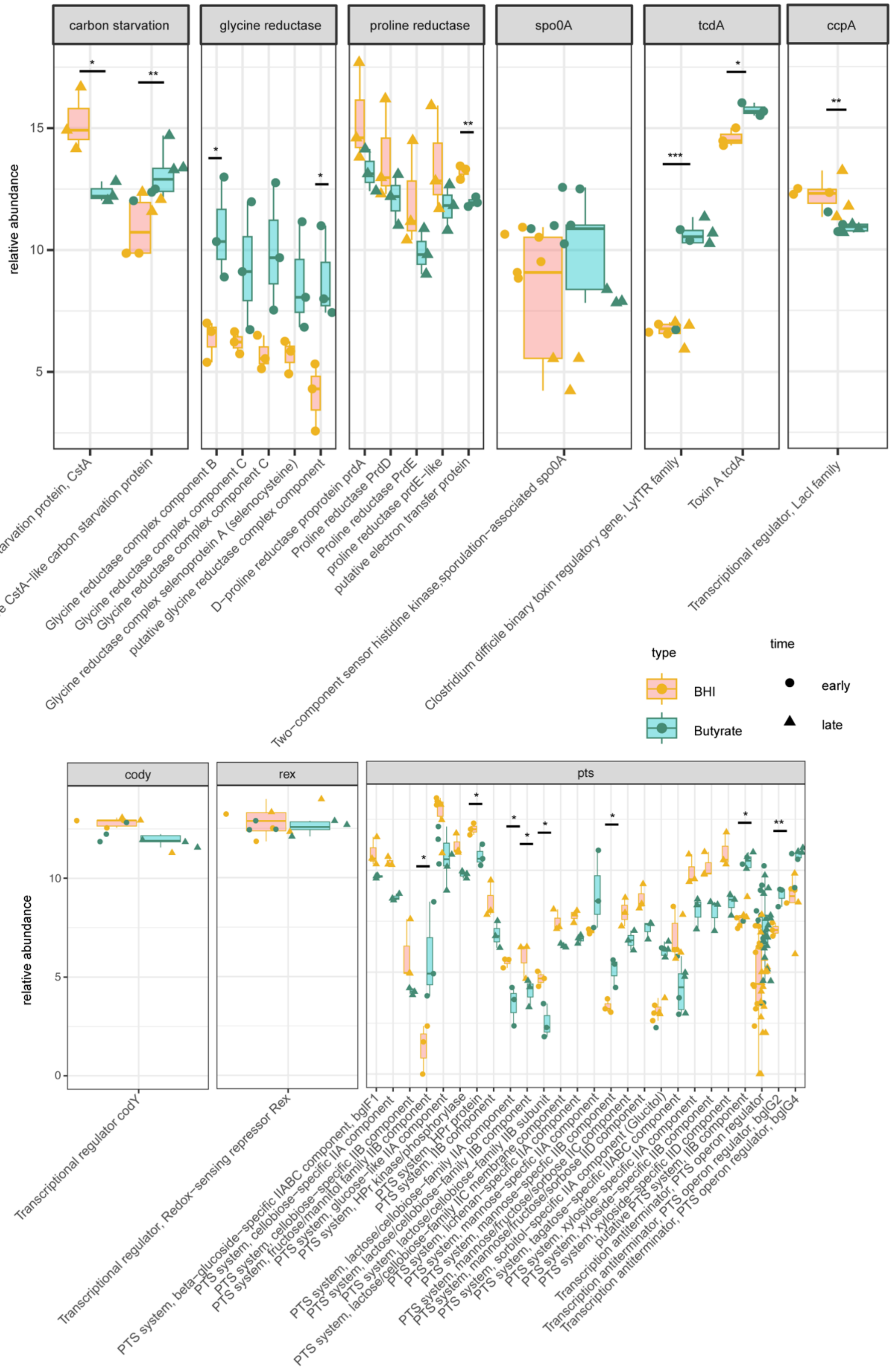

carbon starvation
glycine reductase
proline reductase
spo0A
tcdA
ccpA
relative abundance
15
10
5
*
**
*
*
**
***
*
**
Carbon starvation protein, CstA
putative CstA−like carbon starvation protein
Glycine reductase complex component B
Glycine reductase complex component C
Glycine reductase complex component C
Glycine reductase complex selenoprotein A (selenocysteine)
putative glycine reductase complex component
D−proline reductase proprotein prdA
Proline reductase PrdD
Proline reductase PrdE
proline reductase prdE−like
putative electron transfer protein
Two−component sensor histidine kinase,sporulation−associated spo0A
Clostridium difficile binary toxin regulatory gene, LytTR family
Toxin A tcdA
Transcriptional regulator, LacI family
type
BHI
Butyrate
time
early
late
cody
rex
pts
relative abundance
10
5
0
Transcriptional regulator codY
Transcriptional regulator, Redox−sensing repressor Rex
PTS system, beta−glucoside−specific IIABC component, bglF1
PTS system, cellobiose−specific IIA component
PTS system, cellobiose−specific IIB component
PTS system, fructose/mannitol family IIB component
PTS system, glucose−like IIA component
PTS system, HPr kinase/phosphorylase
PTS system, HPr protein
PTS system, IIB component
PTS system, lactose/cellobiose−family IIA component
PTS system, lactose/cellobiose−family IIB component
PTS system, lactose/cellobiose−family IIB subunit
PTS system, lactose/cellobiose−family IIC membrane component
PTS system, lichenan−specific IIA component
PTS system, mannose−specific IIB component
PTS system, mannose/fructose/sorbose IIC component
PTS system, mannose/fructose/sorbose IID component
PTS system, sorbitol−specific IIA component (Glucitol)
PTS system, tagatose−specific IIABC component
PTS system, xyloside−specific IIA component
PTS system, xyloside−specific IIB component
PTS system, xyloside−specific IID component
putative PTS system, IIB component
Transcription antiterminator, PTS operon regulator
Transcription antiterminator, PTS operon regulator, bglG2
Transcription antiterminator, PTS operon regulator, bglG4

Figure S5. Butyrate modulates select *C. difficile* carbohydrate metabolism and virulence regulators. Relative abundance of normalized transcript counts of select significant genes in early- and late-log. Normalized transcripts were obtained using nnTransform in DESeq. The significance was calculated by one-way ANOVA between transcripts from BHI growth with or without butyrate *p-value < 0.05; **p-value < 0.01; ***p-value < 0.001
